# Clinical Characteristics and Surgical Outcomes of Metastatic Spine Tumors in the Very Elderly: A Prospective Cohort Study in a Super-Aged Society

**DOI:** 10.3390/jcm12144747

**Published:** 2023-07-18

**Authors:** Yutaro Kanda, Kenichiro Kakutani, Yoshitada Sakai, Kunihiko Miyazaki, Tomoya Matsuo, Takashi Yurube, Yoshiki Takeoka, Hiroki Ohnishi, Masao Ryu, Naotoshi Kumagai, Kohei Kuroshima, Yoshiaki Hiranaka, Teruya Kawamoto, Hitomi Hara, Yuichi Hoshino, Shinya Hayashi, Toshihiro Akisue, Ryosuke Kuroda

**Affiliations:** 1 Department of Orthopaedic Surgery, Kobe University Graduate School of Medicine, Kobe 650-0017, Japan; kakutani@med.kobe-u.ac.jp (K.K.); miya625819@gmail.com (K.M.); t.matsuo512@gmail.com (T.M.); takayuru-0215@umin.ac.jp (T.Y.); yoshiki_tkk@hotmail.com (Y.T.); o0717ooo@yahoo.co.jp (H.O.); smart_thomas0724@yahoo.co.jp (M.R.); kumagaiisumi19891107@gmail.com (N.K.); kohei_kuroshima@yahoo.co.jp (K.K.); yoshiagain@gmail.com (Y.H.); trykwmt@med.kobe-u.ac.jp (T.K.); mitohi@med.kobe-u.ac.jp (H.H.); yuichi-h@mta.biglobe.ne.jp (Y.H.); s11793290@yahoo.co.jp (S.H.); kurodar@med.kobe-u.ac.jp (R.K.); 2 Division of Rehabilitation Medicine, Kobe University Graduate School of Medicine, Kobe 650-0017, Japan; yossie@med.kobe-u.ac.jp; 3 Department of Rehabilitation Science, Kobe University Graduate School of Health Sciences, Kobe 654-0142, Japan; akisue@med.kobe-u.ac.jp

**Keywords:** spinal metastases, metastatic tumors, palliative surgery, performance status, activities of daily living, quality of life, spine, very elderly, advanced age, extreme age

## Abstract

The number of advanced-age patients with spinal metastases is rising. This study was performed to clarify the characteristics and surgical outcomes of spinal metastases in advanced-age patients. We prospectively analyzed 216 patients with spinal metastases from 2015 to 2020 and divided them into three age groups: <70 years (*n* = 119), 70–79 years (*n* = 73), and ≥80 years (*n* = 24). Although there were no significant intergroup differences in preoperative characteristics and surgery-related factors except for age, patients aged ≥80 years tended to have a worse performance status (PS), Barthel index, and EuroQol-5 dimension (EQ-5D) before and after surgery than the other two groups. Although the median PS, mean Barthel index and mean EQ-5D greatly improved postoperatively in each group, the median PS and mean Barthel index at 6 months and the mean EQ-5D at 1 month postoperatively were significantly poorer in the ≥80-year group than the 70–79-year group. The rates of postoperative complications and re-deterioration of the EQ-5D were significantly higher in the oldest group than in the other two groups. Although surgery for spinal metastases improved the PS, Barthel index, and EQ-5D regardless of age, clinicians should be aware of the poorer outcomes and higher complication rates in advanced-age patients.

## 1. Introduction

With the recent progress in oncologic treatments and detection technologies, the incidence and prevalence of bone metastases in advanced-age populations have been rising. The estimated incidence of spinal metastases is 30% to 50% in patients with cancer [1]. In approximately 10% to 20% of patients with spinal metastases, the metastases involve spinal element destruction and epidural compression [2], leading to intractable pain, pathologic fractures, or spinal cord compression. All of these factors can significantly deteriorate a patient’s performance status (PS), activities of daily living (ADL), and quality of life (QOL) [3,4,5,6,7,8]. These subjective health values are indispensable indicators, even in the terminal phase as well as objective indicators.

In the management of symptomatic spinal metastases, current evidence has shown that spine surgery with radiotherapy can improve patients’ ambulatory status [9,10] and health status indexes, including the PS, ADL, and QOL for at least 3 to 6 months after surgery [3,4,5,6,7,8]. However, a retrospective matched-pair analysis demonstrated no significant differences in clinical outcomes between radiotherapy plus surgery and radiotherapy alone [11]. Additionally, a more recent study showed that surgery led to an early improvement in motor function but non-significantly better long-term control [12]. This discrepancy may have occurred because some patients with spinal metastases, who are at high risk of surgical complications or a short remaining lifespan, experience insufficient improvement or subsequent deterioration after surgical improvement.

Advanced age is a risk factor for poor outcomes after spine surgery [13,14,15]. People of advanced age are becoming an increasingly prevalent demographic in the Western world, and Japan is the leading super-aged society. Therefore, it becomes increasingly important to clarify the clinical characteristics and surgical outcomes in advanced-aged patients with spinal metastases. The effects of age on outcomes in patients with spinal metastases are still controversial. Furthermore, nearly all prior investigations regarding spinal metastases in advanced-age populations have been limited by a retrospective design. Our previous prospective study of 101 patients demonstrated an improvement in the PS, ADL, and QOL in 65 patients aged <70 years as well as 36 patients aged ≥70 years, although patients aged ≥70 years were likely to experience a re-deterioration of their QOL [4]. However, an analysis of very advanced-aged patients was not performed. Few studies have demonstrated the influence of very advanced age (i.e., older than 80 years) on surgical outcomes in patients with spinal metastases. We thus designed the present prospective study to clarify the clinical characteristics and outcomes following surgery for spinal metastases in patients aged ≥80 years compared with patients aged <70 years and 70 to 79 years.

## 2. Materials and Methods

### 2.1. Ethics Statement

This prospective cohort study was approved by the ethics committee and institutional review board of our hospital (No. 1733; approval date, 6 September 2015). Written informed consent was obtained from each patient. This study was conducted in accordance with the principles of the Declaration of Helsinki and with the laws and regulations of our country.

### 2.2. Patients and Procedures

In total, 264 consecutive patients with spinal metastases, detected from 2015 to 2020, who had an indication for surgery in our hospital, were prospectively enrolled. The patients were divided into three groups based on age: <70 years, 70 to 79 years, and ≥80 years. The indications for surgery were progressive neurological deficits, spinal instability (Spinal Instability Neoplastic Score, SINS [16] of ≥7), or intractable pain refractory to conservative care, including any opioids. Metastatic tumors were diagnosed by plain radiography, computed tomography, magnetic resonance imaging, bone scintigraphy, positron emission tomography, and histological evaluation of samples taken from a needle biopsy or surgery. When a tumor did not originate from a biopsy site and had no identifiable primary site, the tumor was diagnosed as a spinal metastasis from an unknown primary tumor [17]. Emergency surgery was performed for patients who underwent surgery within 48 h of diagnosis because of rapidly progressive neurological dysfunction except for complete paraplegia for >48 h. We excluded patients with dementia or impaired consciousness who were unable to make decisions and accurately evaluate their subjective health state and patients with oligometastasis who underwent a total en bloc spondylectomy for curative treatment. The surgeon carefully chose the surgical procedure based on the neurological function, degree of spinal cord compression, and SINS. Generally, patients with instability (SINS ≥ 7) and tumor-induced spinal canal stenosis with neurological dysfunction underwent posterior instrumentation with decompression. Patients with instability (SINS ≥ 7) and intact neurological function underwent posterior instrumentation without decompression. A few patients without apparent instability (SINS ≤ 6) underwent posterior instrumentation with decompression to prevent future instability from the progression of residual tumor post-laminectomy. All surgeries involved single-stage posterior stabilization with fixation using lateral mass screws for the cervical spine and pedicle screws for the thoracic and lumbar spine. Decompression was achieved with partial removal of the tumor to a feasible extent from the posterolateral aspect. To avoid massive bleeding, neither a corpectomy nor an anterior approach was performed. No preoperative embolization was performed. All immobilization devices, including corsets and collars, were removed after surgery because the instrumentation provided sufficient spinal stability. The treatment plan was determined by a multidisciplinary team focused on bone metastases. Postoperative radiotherapy and/or chemotherapy were performed after the surgical site was closed at 2 weeks postoperatively. If indicated, percutaneous vertebroplasty was performed as a postoperative salvage procedure for an adjacent vertebral fracture or as a palliative treatment for patients who were considered unable to tolerate surgery.

### 2.3. Evaluation

The postoperative survival duration was defined as the time from the date of surgery to the latest follow-up examination or death. At the start of the study (baseline), we registered the age, sex, and primary tumor type as preoperative demographic factors. The new Katagiri score [18] and revised Tokuhashi score [19] were also recorded. The part of the new Katagiri score pertaining to the primary lesion was used to evaluate the malignancy grade of the primary tumor (slow/moderate/rapid growth) [18]. As surgery-related factors, we investigated the operative time, blood loss, number of fused vertebrae, screw technique (open or percutaneous), and postoperative complications (defined as Clavien–Dindo grade ≥2) [20]. Given the nature of surgery for spinal metastases, the grade 2 blood transfusion was not included as a complication. The Eastern Cooperative Oncology Group PS (ECOGPS) [21], Barthel index [22], and EuroQol-5 Dimension (EQ-5D) [23] were used to evaluate the PS, ADL, and QOL, respectively. Neurological function was assessed with the Frankel grade [24]. Clinical follow-up was routinely performed at 1, 3, and 6 months postoperatively and then every 3 months. We collected follow-up data until 31 December 2021. Improvement and deterioration of the PS and QOL were defined as a ≥1-level change in the PS, a ≥10-point change in the Barthel index, and a ≥10% change in the EQ-5D score, respectively. Based on these definitions, the improvement rate, unchanged rate, and deterioration rate within 6 months were calculated. Re-deterioration was defined as a subsequent deterioration within 6 months after one-time improvement or maintenance. Patients who were alive and could not consult our department were contacted by telephone to obtain the latest follow-up information.

### 2.4. Statistical Analysis

All statistical analyses were performed using IBM SPSS Statistics 28.0 (IBM Corp., Armonk, NY, USA). The overall survival rate was calculated by the Kaplan–Meier method, and the three groups were compared by the Cox model. One-way analysis of variance with the Tukey–Kramer post hoc test was used to compare the continuous variables among the demographics and clinical characteristics at baseline between patients aged <70 years, 70 to 79 years, and ≥80 years. The chi-squared test was used to compare the categorical variables at baseline among the three groups. For the chronological evaluation, the Kruskal–Wallis test and Bonferroni post hoc test were used to assess the significance of differences among the three groups. The improvement rate, deterioration rate, and re-deterioration rate of the subjective health values were compared among the three groups using the chi-squared test and residual analysis. The statistical significance was set at *p* < 0.05.

## 3. Results

### 3.1. Patient Demographics and Clinical Characteristics at Surgery

**Figure 1:**
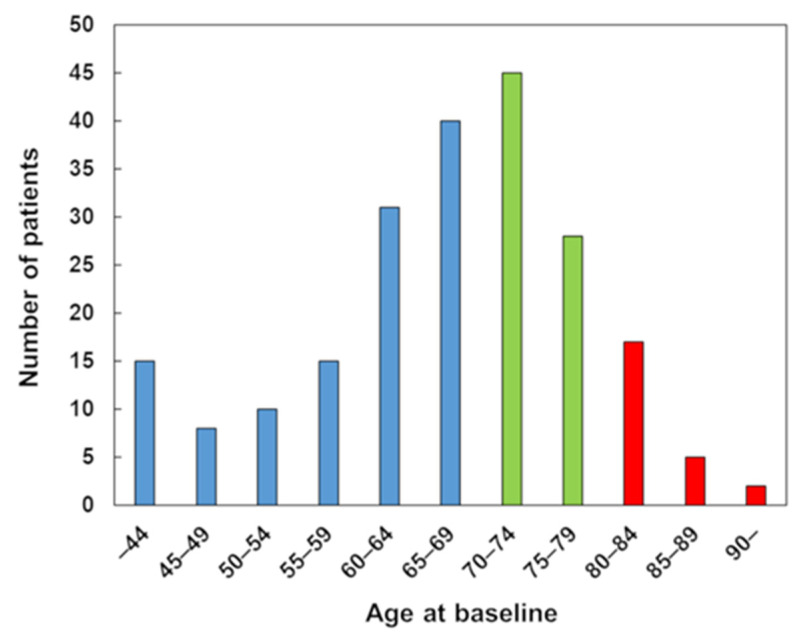
Age distribution at baseline. Blue, green, and red bars indicate patients aged <70 years, 70–79 years, and ≥80 years, respectively.

In total, 216 patients were analyzed (mean age, 66.4 years; range 24–92 years), comprising 119 patients aged <70 years, 73 aged 70 to 79 years, and 24 aged ≥80 years. The age distribution is shown in Figure 1. Thirty-eight patients were excluded because they declined surgery, four patients were excluded because of impaired consciousness, and three patients were excluded because they underwent a total en bloc spondylectomy due to oligometastasis. No patients were lost to follow-up before death. Thus, the patients who did not die within 6 months postoperatively were monitored for at least 6 months. There were no significant differences in clinical characteristics at surgery or surgery-related factors (including the surgical method and screw technique) among the three groups except for age (*p* < 0.001); patients aged ≥80 years had a tendency to have a worse EQ-5D score at surgery (Table 1). Although there was no significant difference in malignancy according to the new Katagiri score (slow/moderate/rapid growth) between the three groups, the most common primary type of cancer differed by age; lung cancer was the most common type of cancer in patients aged <70 years (21.8%) and 70 to 79 years (17.8%), whereas colon cancer was the most common cancer in patients aged ≥80 years (20.8%) (Table 2).

### 3.2. Survival Rate

**Figure 2:**
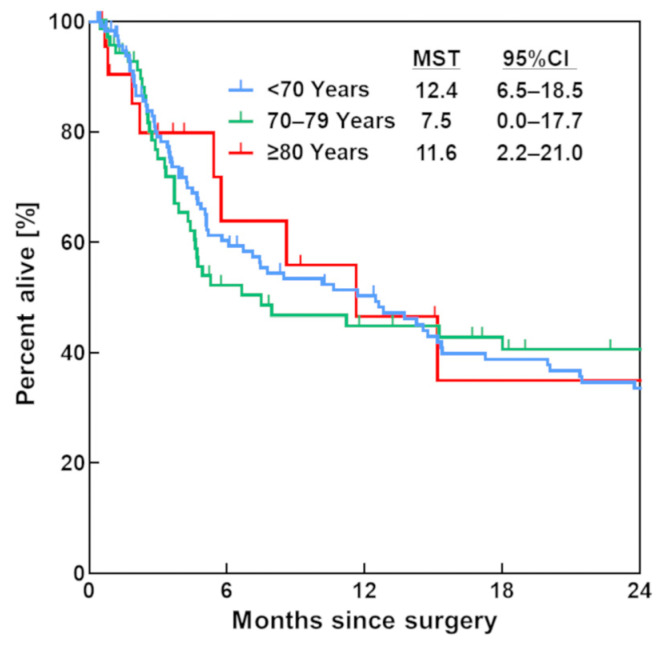
Kaplan–Meier survival curve. Blue, green, and red lines indicate patients aged <70 years, 70–79 years, and ≥80 years, respectively. MST, median survival time; CI, confidence interval.

The median survival time after surgery was 12.4 months (95% confidence interval (CI), 6.5–18.5) in patients aged <70 years, 7.5 months (95% CI, 0.0–17.7) in patients aged 70 to 79 years, and 11.6 months (95% CI, 2.2–21.0) in patients aged ≥80 years (Figure 2). There was no significant difference in survival time among the three groups (*p* = 0.730) (Figure 2).

### 3.3. Complications

A total of 47 postoperative complications occurred in 40 (18.5%) of the 216 patients. Wound infection and/or dehiscence were the most common (13 patients), followed by pneumonia (7 patients). There was a significant difference in the rate of complications between the three groups (*p* = 0.036). The residual analysis showed that the rate of postoperative complications was significantly higher in patients aged ≥80 years (37.5%) than that in patients aged 70 to 79 years (17.8%) (*p* = 0.023). Pneumonia developed in four (16.7%) of the 24 patients aged ≥80 years (including two cases of aspiration pneumonia), two (1.7%) of the 119 patients aged <70 years, and one (1.4%) of the 73 patients aged 70 to 79 years. No aspiration pneumonia occurred in patients under 80 years of age (Table 3).

### 3.4. ECOGPS (PS)

**Figure 3:**
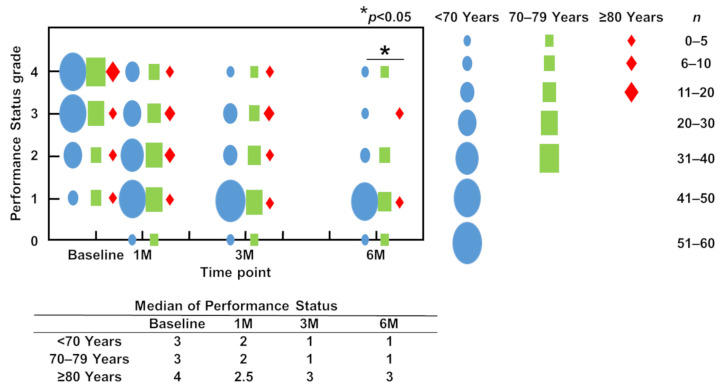
Performance status preoperatively and at 1, 3, and 6 months postoperatively. Blue circles, green squares, and red diamonds indicate the number of patients aged <70 years, 70–79 years, and ≥80 years, respectively. Data were assessed using the chi-squared test. * *p* < 0.05.

The median PS at surgery was 3 in patients aged <70 years and 70 to 79 years and 4 in patients aged ≥80 years (Figure 3). At 1 month postoperatively, the PS had improved to 2 in patients aged <70 years and 70 to 79 years and to 2.5 in patients aged ≥80 years. In patients aged <70 years and 70 to 79 years, the PS further improved to 1 at 3 months postoperatively; this improvement was maintained until 6 months postoperatively. However, the PS deteriorated in patients aged ≥80 years to 3 at both 3 and 6 months postoperatively. There was a significant difference in the PS at 6 months postoperatively among the three groups (*p* < 0.001) (Figure 3).

### 3.5. Barthel Index (ADL)

**Figure 4:**
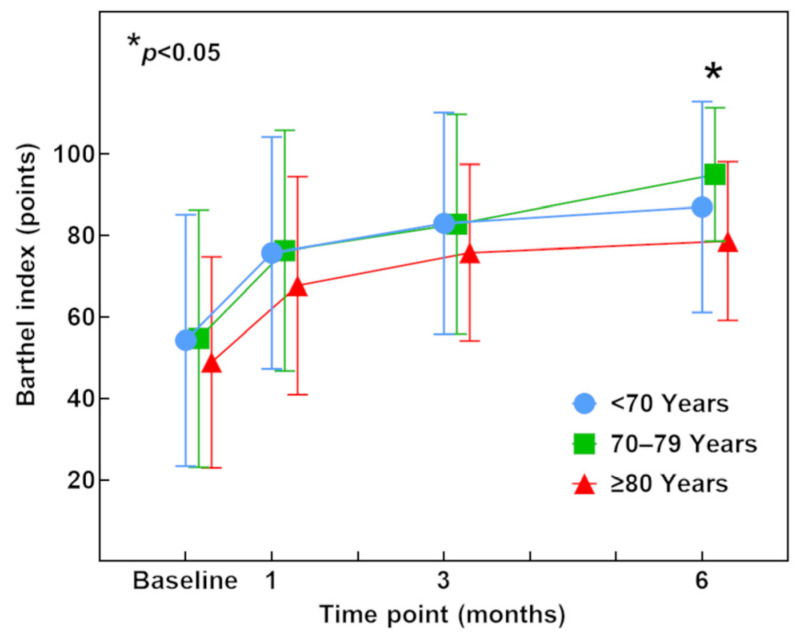
Barthel index preoperatively and at 1, 3, and 6 months postoperatively. Blue, green, and red lines indicate patients aged <70 years, 70–79 years, and ≥80 years, respectively. Data were assessed using the Kruskal–Wallis test and Bonferroni post hoc test. * *p* < 0.05 between patients aged 70–79 years and ≥80 years at 6 months postoperatively.

The mean Barthel index at surgery was 54.3 (range 0–100) in patients aged <70 years, 54.7 (range 0–100) in patients aged 70–79 years, and 48.9 (range 10–100) in patients aged ≥80 years (Table 1). At 1 month postoperatively, the mean Barthel index had greatly improved to 75.7 (range 0–100), 76.3 (range 0–100), and 67.7 (range 0–100), respectively, in the <70-year, 70–79-year, and ≥80-year age groups. At 3 and 6 months postoperatively, the mean Barthel index further improved in each group. The chronological course of the Barthel index tended to be lower in patients aged ≥80 years than in the other two age groups throughout the first 6 months postoperatively, and the difference between patients aged 70–79 years and ≥80 years reached statistical significance at 6 months postoperatively (*p* = 0.048) (Figure 4).

### 3.6. EQ-5D Score (QOL)

**Figure 5:**
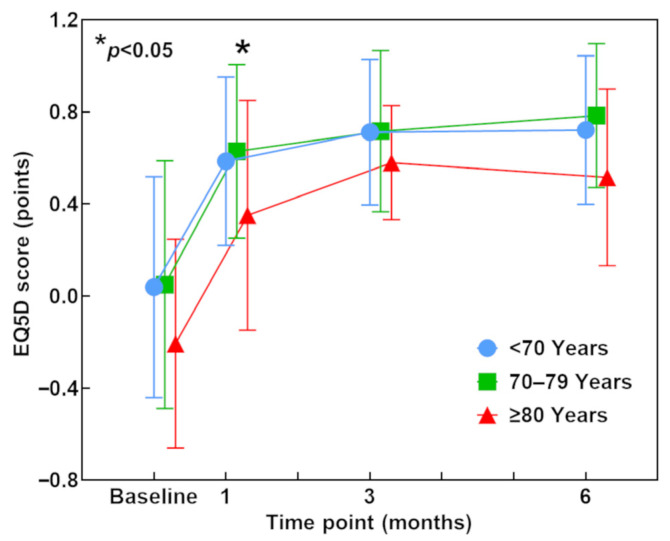
EQ-5D score preoperatively and at 1, 3, and 6 months postoperatively. Blue, green, and red lines indicate patients aged <70 years, 70–79 years, and ≥80 years, respectively. Data were assessed using the Kruskal–Wallis test and Bonferroni post hoc test. * *p* < 0.05 between patients aged 70–79 years and ≥80 years at 1 month postoperatively. EQ-5D, EuroQol-5 Dimension.

The mean EQ-5D score at surgery was 0.004 (range −0.594–1.000) in patients aged <70 years, 0.049 (range −0.594–1.000) in patients aged 70 to 79 years, and −0.207 (range −0.594–0.691) in patients aged ≥80 years (Table 1). At 1 month postoperatively, the mean EQ-5D score had greatly improved to 0.586 (range −0.594–1.000), 0.629 (range −0.331–1.000), and 0.351 (range −0.594–0.814), respectively, in the <70-year, 70–79-year, and ≥80-year age groups. At 3 and 6 months postoperatively, the mean EQ-5D further improved in each group. The chronological course of the EQ-5D score tended to be lower in patients aged ≥80 years than in the other two age groups throughout the first 6 months postoperatively, and the difference between patients aged 70–79 years and ≥80 years reached statistical significance at 1 month postoperatively (*p* = 0.039) (Figure 5).

### 3.7. Individual Chronological Changes

In addition to the overall transition in each group, we also analyzed the outcomes from each patient’s viewpoint. There was no significant difference in the improvement, unchanged, and deterioration rates of the ECOGPS, Barthel index, and EQ-5D score among the three groups. The re-deterioration rates of the ECOGPS (20.8%) and Barthel index (21.2%) tended to be higher in patients aged ≥80 years than in patients aged <70 years and 70 to 79 years, although the differences did not reach statistical significance (*p* = 0.486 and 0.073, respectively). The re-deterioration rate of the EQ-5D score significantly differed between age groups (*p* = 0.049) and was 34.8% in patients aged ≥80 years, 13.7% in patients aged <70 years, and 17.1% in patients aged 70 to 79 years (Table 4). The residual analysis showed that the rate of re-deterioration of the EQ-5D was significantly higher in patients aged ≥80 years than in patients aged 70 to 79 years (*p* = 0.016).

## 4. Discussion

Our prospective cohort study of 216 patients with spinal metastases demonstrated that even patients aged ≥80 years achieved great improvements in their PS, ADL, and QOL after surgery. However, patients aged ≥80 years showed a relatively poorer PS, ADL, and QOL before and after surgery compared with the other groups. From the patients’ perspectives, re-deterioration of QOL was highest in the oldest group. As patients aged ≥70 years had a higher re-deterioration rate than those aged <70 years in our previous study [4], this finding contributes to more conclusive evidence regarding the effect of age on the outcomes of surgery for spinal metastases. In addition, patients aged ≥80 years had a higher risk of postoperative complications and re-deterioration of QOL than the other two age groups, although there was no significant difference between patients aged <70 years and ≥70 years in our previous study [4]; these findings may more accurately reflect the effects of advanced age on surgical outcomes for spinal metastases. Patients aged ≥80 years rather than ≥70 years might be considered a true advanced-age group in spinal metastases surgery.

Our findings revealed that although patients aged ≥80 years achieved great improvements in their PS, ADL, and QOL after spinal surgery, they had poorer values compared with patients under 80 years of age. Limited evidence regarding the effects of advanced age on surgical outcomes for spinal metastases is currently available. A retrospective study of 92 patients aged ≥60 years showed that spinal surgery can achieve pain relief in 90% of patients and improvement in the Karnofsky PS in 63% of patients [25]. However, it is imperative to reflect on our aging society and evaluate the clinical outcomes in more advanced-age patients. In fact, approximately one-third of our cohort comprised patients aged from 70 to 79 years, and one-ninth of patients were ≥80 years. A retrospective multicenter study demonstrated that symptomatic recovery after decompression surgery for lumbar spinal stenosis was similar between 46 patients aged ≥80 years and 195 patients aged <80 years [26]. In addition, a multicenter study showed a significant benefit of surgery for lumbar spinal stenosis over nonoperative treatment in 105 patients aged ≥80 years [27]. In contrast, a retrospective multicenter study of 77 patients aged ≥80 years showed that posterior decompression surgery for cervical myelopathy is beneficial for patients aged ≥80 years, despite providing a more limited neurological recovery compared with younger patients [28]. Although our recent study of 36 patients aged ≥70 years demonstrated favorable outcomes of spinal surgery in terms of the ECOGPS, Barthel index, and EQ-5D score compared with patients aged <70 years [4], there is still concern regarding whether spinal surgery improves patients’ PS, ADL, and QOL, even in patients aged ≥80 years and in those undergoing other spine surgeries. Therefore, the improvements provided by surgery in the present study cohort will be helpful for clinicians when selecting treatment strategies for patients aged ≥80 years with spinal metastases.

The PS, ADL, and QOL at the time of surgery were relatively worse in patients aged ≥80 years than in the other two groups. Although this might have impacted the postoperative course, the PS, ADL, and QOL were also poorer throughout the first 6 months postoperatively in patients aged ≥80 years than in younger patients. Early multidisciplinary interventions before the development of severe symptoms could ameliorate their outcomes, although natural deterioration of QOL with age might have partly contributed to these results. We performed an additional analysis from each patient’s viewpoint as a more detailed evaluation. Although changes in each patient are important, few studies have focused on patients’ viewpoints. Patients aged ≥80 years showed a trend toward subsequent deterioration within 6 months after a one-time improvement or maintenance of the PS, ADL, and QOL. In particular, the difference between age groups in the re-deterioration rate of QOL reached statistical significance. These results are consistent with a prior report showing a higher re-deterioration rate of QOL in patients aged ≥70 years [4]. Interestingly, patients aged ≥80 years exhibited a significantly higher rate, even when compared with those aged 70 to 79 years. Given that there was no significant difference in the overall survival and degree of malignancy between the three groups, the higher re-deterioration rate within 6 months in patients aged ≥80 years would likely be due to the differences in age-related characteristics rather than their disease progression. Therefore, surgeons should keep these findings in mind when making decisions regarding their treatment strategies for advanced-age patients with spinal metastases.

Our results also suggest that patients aged ≥80 years are at higher risk of complications (37.5%) than patients aged <80 years, despite the similar complication rate between patients aged ≥70 years and <70 years in our previous study [4]. In fact, this rate is higher than the complication rates in previous reports that included all age groups (range 20%–34%) [29,30,31]. Amelot et al. [32] reported that postoperative complications occurred in 17 (33.3%) of the 51 patients aged ≥80 years, which was significantly higher than the rate in younger patients. This result is consistent with ours; however, the breakdown was slightly different. The main complications in the study by Amelot et al. [32] were wound complications, whereas the main complication in our study was pneumonia, including aspiration pneumonia. In older patients, it might be useful to provide interventions by speech-language pathologists and optimize the types of food to prevent postoperative pneumonia.

This study has several limitations. First, the number of patients aged ≥80 years was relatively small, although the proportion was higher (11.1%) than in a prior report (3.7%) [32]. As the aging of society progresses, it is necessary to perform a multicenter prospective study with a larger number of patients aged ≥80 years. Second, whether patients selected surgery also depended on the patient’s willingness; consequently, selection bias may exist. Patients aged ≥80 years with lower vitality might be reluctant to select surgery, although there were no significant differences in the preoperative characteristics among the three groups. Third, there is still room for improvement in selecting the preoperative procedure(s). Preoperative embolization is advantageous in reducing bleeding [33]. We did not perform preoperative embolization in our cohort, and a few patients had massive bleeding.

In conclusion, surgery for spinal metastases improved the PS, ADL, and QOL, regardless of age. However, it should be noted that patients aged ≥80 years have relatively poorer clinical outcomes both before and after surgery and higher rates of complications and re-deterioration of QOL compared with younger patients. Patients aged ≥80 years rather than ≥70 years might be considered a true advanced-age group in spinal metastases surgery. Although advanced age is not a contraindication to surgery, careful patient selection may be needed. Early multidisciplinary interventions before the development of severe symptoms could ameliorate their outcomes.

## Figures and Tables

**Table 1:** Demographics and clinical characteristics of patients at surgery according to age.

	<70 Years	70–79 Years	≥80 Years	
	*n* = 119	*n* = 73	*n* = 24	*p*
Age (mean (range))	58.2 (24–69)	73.8 (70–79)	84.1 (80–92)	<0.001
Sex (*n*)				0.945 *
Male	71	45	15	
Female	48	28	9	
Malignancy of primary cancer				0.157 *
Slow growth	25	22	4	
Moderate growth	56	24	8	
Rapid growth	38	27	12	
New Katagiri score (mean (range))	5.3 (1–9)	5.1 (1–8)	5.0 (1–8)	0.725
Revised Tokuhashi score (mean (range))	5.8 (1–13)	6.3 (1–11)	6.3 (1–12)	0.366
SINS (mean (range))	10.5 (3–18)	10.4 (4–16)	11.3 (2–17)	0.399
Lesion location				0.539 *
Cervical spine	19	14	3	
Thoracic spine	73	44	11	
Lumbar spine	24	13	9	
Sacral spine	3	2	1	
Preoperative chemotherapy (*n*)				0.438 *
Yes	51	38	12	
No	68	35	12	
Preoperative radiotherapy (*n*)				0.407 *
Yes	35	22	4	
No	84	51	20	
ECOGPS grade (*n*)				0.206 *
PS 1	8	8	1	
PS 2	23	9	2	
PS 3	41	25	5	
PS 4	47	31	16	
Barthel index (mean (range))	54.3 (0–100)	54.7 (0–100)	48.9 (0–100)	0.711
EQ-5D (mean (range))	0.004 (−0.594–1.000)	0.049 (−0.594–1.000)	−0.207 (−0.594–0.691)	0.092
Frankel classification (*n*)				0.496 *
Garde A, B, and C	47	35	11	
Garde D and E	72	38	13	
Surgical method				0.725 *
Instrumentation and decompression	94	60	18	
Instrumentation alone	25	13	6	
Surgery type (*n*)				0.174 *
Emergency surgery	43	23	4	
Non-emergency surgery	76	50	20	
Screw technique (*n*)				0.166 *
Open technique	87	57	14	
Percutaneous technique	32	16	10	
Operative time (mean (range)) (min)	190.9 (59–440)	196.2 (30–370)	176.1 (73–370)	0.464
Blood loss (mean (range)) (g)	317.3 (0–2500)	371.3 (0–1800)	269.9 (0–820)	0.441
Number of fused vertebrae (mean (range))	6.3 (3–12)	6.5 (3–12)	6.0 (3–9)	0.927

* Assessed using the chi-squared test. SINS, Spinal Instability Neoplastic Score; ECOG, Eastern Cooperative Oncology Group; PS, performance status; EQ-5D, EuroQol-5 Dimension.

**Table 2:** Primary tumor types at surgery according to age. Data are presented as *n* (%).

	Total	<70 Years	70–79 Years	≥80 Years
	*n* = 216	*n* = 119	*n* = 73	*n* = 24
Lung	42 (19.4)	26 (21.8)	13 (17.8)	3 (12.5)
Kidney	21 (9.7)	9 (7.6)	9 (12.3)	3 (12.5)
Liver	20 (9.3)	11 (9.2)	7 (9.6))	2 (8.3)
Breast	19 (8.8)	12 (10.1)	4 (5.5)	3 (12.5)
Myeloma	15 (6.9)	10 (8.4)	4 (5.5)	1 (4.2)
Thyroid	13 (6.0)	3 (2.5)	9 (12.3)	1 (4.2)
Lymphoma	13 (6.0)	8 (6.7)	4 (5.5)	1 (4.2)
Colon	13 (6.0)	4 (3.4)	4 (5.5)	5 (20.8)
Prostate	9 (4.2)	5 (4.2)	3 (4.1)	1 (4.2)
Bladder	4 (1.9)	0 (0.0)	3 (4.1)	1 (4.2)
Esophagus	4 (1.9)	3 (2.5)	1 (1.4)	0 (0.0)
Unknown	6 (2.8)	2 (1.7)	4 (5.5)	0 (0.0)
Others	39 (18.1)	26 (21.8)	8 (11.0)	3 (12.5)

**Table 3:** Numbers and rates of postoperative complications according to age.

	Total	<70 Years	70–79 Years	≥80 Years
	*n* = 216	*n* = 119	*n* = 73	*n* = 24
Number of patients (%)	40 (18.5)	18 (15.1)	13 (17.8)	9 (37.5)
Wound infection/dehiscence	13	9	3	1
Pneumonia	7	2	1	4
Urinary tract infection	5	3	0	2
Implant failure	5	2	2	1
Adjacent segment fracture	4	3	1	0
Deep vein thrombosis	3	1	2	0
Others	10	4	4	2
Number of complications	47	24	13	10

**Table 4:** Individual chronological changes in the ECOGPS, Barthel index, and EQ-5D score within 6 months postoperatively according to age. Data are presented as *n* (%). The chi-squared test and residual analysis were used to identify the difference in re-deterioration rates between patients aged <70 years, 70–79 years, and ≥80 years.

	<70 Years	70–79 Years	≥80 Years	
	*n* = 119	*n* = 73	*n* = 24	*p*
ECOGPS				
Improvement	89 (74.8)	55 (75.3)	18 (75.0)	0.928
Unchanged	27 (22.7)	17 (23.3)	6 (25.0)	
Deterioration	3 (2.5)	1 (1.4)	0 (0.0)	
Re-deterioration	16 (13.8)	8 (14.3)	5 (20.8)	0.486
Barthel index				
Improvement	87 (73.1)	50 (68.5)	15 (62.5)	0.175
Unchanged	28 (23.5)	23 (31.5)	7 (29.2)	
Deterioration	4 (3.4)	0 (0.0)	2 (8.3)	
Re-deterioration	11 (14.3)	10 (10.3)	6 (21.2)	0.073
EQ-5D				
Improvement	105 (88.2)	62 (84.9)	18 (75.0)	0.464
Unchanged	12 (10.1)	8 (11.0)	5 (20.8)	
Deterioration	2 (1.7)	3 (4.1)	1 (4.2)	
Re-deterioration	16 (13.7)	12 (17.1)	8 (34.8)	0.049

ECOG, Eastern Cooperative Oncology Group; PS, performance status; EQ-5D, EuroQol-5 Dimension.

## Data Availability

The data presented in this study are available on reasonable request from the corresponding author.
